# A two-armed, randomised, controlled exploratory study of adding the AmbuGard cleaning system to normal deep-cleaning procedures in a regional ambulance service

**DOI:** 10.29045/14784726.2020.09.5.2.10

**Published:** 2020-09-01

**Authors:** Graham McClelland, Karl Charlton, Jacqueline Mains, Karen Millican, Caroline Cullerton

**Affiliations:** North East Ambulance Service NHS Foundation Trust ORCID iD: https://orcid.org/0000-0002-4502-5821; North East Ambulance Service NHS Foundation Trust; North East Ambulance Service NHS Foundation Trust; North East Ambulance Service NHS Foundation Trust; Freeman Hospital

**Keywords:** ambulance, cleaning, infection

## Abstract

**Background:**

Ambulance services transport patients with infections and diseases, and could pose a cross-transmission risk to patients and staff through environmental contamination. The literature suggests that environmental pathogens are present in ambulances, cleaning is inconsistent and patient/staff impact is difficult to quantify. Eco-Mist developed a dry misting decontamination system for ambulance use called AmbuGard, which works in < 30 minutes and is 99.9999% effective against common pathogens. The research question is: ‘What pathogens are present in North East Ambulance Service ambulances and what impact does adding AmbuGard to the deep-cleaning process make?’.

**Methods:**

A two-armed, randomised controlled trial enrolled 14 ambulances during their regular 24-week deep clean, which were 1:1 randomised to deep cleaning (control arm) or deep cleaning plus AmbuGard (intervention arm). Polywipe swabs were taken before and after cleaning from five locations selected for high rates of contact (steering wheel, shelf, side-door grab rail, patient seat armrest, rear door handle/grab rail). Microbiology culture methods identified the presence and amount of bacterial organisms present, including the selected pathogens: *Enterococcus* spp.; *Enterobacter* spp.; *Klebsiella* spp.; *Staphylococcus aureus*; *Acinetobacter* spp.; *Pseudomonas* spp.; *Clostridium difficile*; coagulase-negative staphylococci (CoNS). The researcher taking the swabs and the laboratory were blinded to the trial arm.

**Results:**

Pathogens of interest were found in 10 (71%) vehicles. CoNS were found in all vehicles. Pathogens were found on all locations swabbed. Normal deep cleaning was effective at eliminating pathogens and the addition of AmbuGard showed no obvious improvement in effectiveness.

**Conclusion:**

Pathogens associated with healthcare-acquired infections were found throughout all ambulances. Normal deep cleaning was effective, and adding AmbuGard showed no obvious improvement. This was a small study at a single point in time. Further research is needed into temporal trends, how to reduce pathogens during normal clinical duties and patient/staff impact.

## Introduction and background

Ambulance services treat and transport patients with a variety of known and unknown infections, diseases and conditions. Patients include those who are particularly vulnerable to contracting infections due to extremes of age, being immunosuppressed or having concurrent illnesses or injuries. Clinicians who work on ambulances are exposed to a range of pathogens and risk contracting, or passing on, illnesses. Ambulance staff (includes all staff employed by an ambulance service) reported a 5.5% sickness rate in 2015/2016, which is higher than the NHS average (National Audit Office, 2017).

The National Patient Safety Agency has recommended that each ambulance trust have procedures in place to ensure cleanliness, but their guidance leaves it up to individual trusts to interpret and apply this framework. There is a requirement for monitoring and audit within the framework, but little prehospital evidence to underpin any of the recommendations (National Patient Safety Agency, 2009).

A study initiated by the National Ambulance Service Infection Prevention and Control Group found variations in cleaning practice between UK ambulance services. One finding from the report was that nationally the average % of swabs showing heavy contamination (> 100 Relative Light Units measured using adenosine triphosphate (ATP) swabs) as a measure of viable microbial organisms was 12.4% (currently unpublished). A study conducted in Yorkshire Ambulance Service (YAS) explored the impact of an ambulance vehicle preparation service (make ready crew) using ATP swabbing, and showed that make ready crews outperformed normal cleaning practices (Mackenzie & Pilbery, 2019).

In one of few UK-based studies in this area, Nigam and Cutter (2003) described bacteria that were found on Welsh ambulances both before and after cleaning. More recently the SEKURE study (Wepler et al., 2015) described how Methicillin-resistant *Staphylococcus aureus* (MRSA) was found in German ambulances, how patient contact areas were the most frequently contaminated sites and how effective cleaning was difficult. MRSA, and other pathogens, have been consistently reported in ambulances all around the world (Alrazeeni & Al Sufi, 2014; Alves & Bissell, 2008; Eibicht & Vogel, 2011; Galtelli et al., 2006; Lee et al., 2006; Rago et al., 2012; Vikke & Giebner, 2016).

This body of literature, although small, suggests that pathogens are found in ambulances, that cleaning is inconsistent, that the impact on ambulance staff and patients is difficult to quantify and that there is a need for more research in this area.

Eco-Mist Biotechnics has developed a dry misting decontamination system called AmbuGard for the ambulance service market. It is designed to sanitise an ambulance in < 30 minutes, and is 99.9999% effective against most common pathogens using the TriBioSan sanitising solution – a proprietary, stable, hypochlorus acid. AmbuGard is used by some European ambulance services and private ambulance services in the UK, but there have been no studies in an NHS ambulance service and no evidence of clinical effectiveness in an NHS setting (Eco-Mist Biotechnics, 2019).

This project will describe the pathogens linked to healthcare-associated infections (HAIs) found in North East Ambulance Service NHS Foundation Trust (NEAS) ambulances and the effectiveness of deep cleaning, with and without the AmbuGard system, at decontaminating the ambulance.

## Methods

This study was an exploratory two-armed, randomised, controlled study with blinded outcome assessment.

The study was carried out at Pallion Ambulance Station, which houses the NEAS fleet as well as workshops provided by North East Ambulance Service Unified Solutions (NEASUS). Pallion is where all NEAS vehicles are deep cleaned. All front-line emergency ambulances are deep cleaned every six weeks (level 1 deep clean), and every 24 weeks the ambulance is stripped of all removable items to facilitate the deep clean (level 2 deep clean). Prior to the start of the study, the dedicated team of vehicle cleaning staff based at Pallion were trained in the use of the AmbuGard system. Product directions in terms of placement, timings and use of the TriBioSan solution were followed.

A consecutive series of emergency ambulances were selected based on the inclusion/exclusion criteria below:

- Inclusion criteria: ambulance due for 24-week (level 2) deep clean
- Exclusion criteria: rapid response car

The study involved:

- Pre-cleaning polywipe swabs
- Vehicle randomised
- Vehicle deep cleaned +/- AmbuGard
- Post-cleaning polywipe swabs
- Swabs sent to laboratory for analysis

The intervention comprised putting the AmbuGard unit into the vehicle after normal deep cleaning, closing the doors and allowing it to mist the ambulance for 20 minutes. The hatch between the cab and the saloon was left open to allow the mist to circulate.

All swabs were taken by a member of the research team who was blinded as to whether AmbuGard would be, or had been, used. Data were collected from the following predetermined locations in the ambulance, as they had been identified as areas of high patient/staff contact:

- Steering wheel
- Grab rail inside ambulance by side door
- Arm rest nearest centre of vehicle on forward-facing patient seat
- Shelf behind hatch between cab and body of ambulance
- Handles and grab rails inside back door

Ambulances were randomised by the lead author (GM) using a predetermined sequence of sealed envelopes to either: normal cleaning (control arm) or normal cleaning plus AmbuGard (intervention arm). The order that vehicles were allocated to AmbuGard or standard deep cleaning was 1:1 randomised, using a block randomisation sequence generated by RANDOM.ORG.

Pathogens of interest were selected by the NEAS infection and prevention control (IPC) manager based on current concerns in the IPC field and their links to HAIs, and included:

- *Enterococcus*
- *Enterobacter*
- *Klebsiella*
- *Staphylococcus aureus*
- *Acinetobacter*
- coagulase-negative staphylococci (CoNS)
- *Pseudomonas*
- *Clostridium difficile*

Polywipe swabs were delivered to the Microbiology department, who performed conventional selective and non-selective cultures for growth of the target pathogens along with any other bacterial organisms (Table 1). In addition, selective conventional culture and Esculin enrichment culture were performed in parallel for the presence of *C. difficile*. Identification of all organisms was performed using matrix-assisted laser desorption ionisation time-of-flight mass spectrometry. Results from the laboratory reported the presence of each pathogen of interest and the number of colony-forming units (CFUs) that were present. CFUs greater than 100 were reported as > 100.

**Table 1.** Laboratory culture methods.

Media	Atmosphere	Temperature	Incubation time
**CPSE (bioMérieux)**	Aerobic	37^o^C	48 hours
***Colorex Staph aureus* (E&O)**	Aerobic	37^o^C	24 hours
**Blood agar (in-house)**	Aerobic	37^o^C	48 hours
**Blood agar (in-house)**	Aerobic	30^o^C	48 hours
**Blood agar (in-house)**	Anaerobic	37^o^C	48 hours
**chromID *C. difficile* (bioMérieux)**	Anaerobic	37^o^C	48 hours
**Esculin broth (in-house)**	Aerobic	37^o^C	72 hours

After the final vehicle had been cleaned, the cleaning staff involved in the study were asked for feedback using a simple survey.

## Statistics and data analysis

### Sample size calculation

The sample size for this exploratory study was determined by the funding available for laboratory analysis.

### Statistical analysis plan

Descriptive statistics were used to summarise the study data given the small numbers and lack of power.

## Peer review

This study was reviewed within NEAS by the R&D department and externally by the North East & North Cumbria Academic Health Sciences Network (NE&NC AHSN) and the College of Paramedics R&D group, and comments from both groups were incorporated in the study. This project was presented to the North East Research Design Service (RDS), who provided methodological advice. The study was presented to the North East Healthwatch group in April 2019, who were supportive of the idea.

## Results

Fourteen ambulances, seven control and seven intervention, were enrolled into the study over a period of seven weeks (August to October 2019) by four researchers. The median number of days since the vehicles had had their last similar deep clean was 170 (IQR 169–183) days. Table 2 displays the number of vehicles in which each pathogen was reported. Some vehicles had multiple pathogens, so the numbers are not cumulative.

**Table 2.** Vehicles in which pathogens were found, pre and post cleaning.

	Control		AmbuGard	
	Pre clean	Post clean	Pre clean	Post clean
***Enterococcus***	0	0	1	2*
***Enterobacter***	0	0	1	0
***Klebsiella***	0	0	0	0
***S. aureus***	2	0	0	0
***Acinetobacter***	2	0	4	0
***Pseudomonas***	1	0	3	0
***C. difficile***	1	0	0	0
**CoNS**	7	6	7	7
**Other**	7	3	7	3

*The two vehicles where *Enterococcus* was found post clean were different to the vehicle where it was found pre clean.

**Figure 1:**
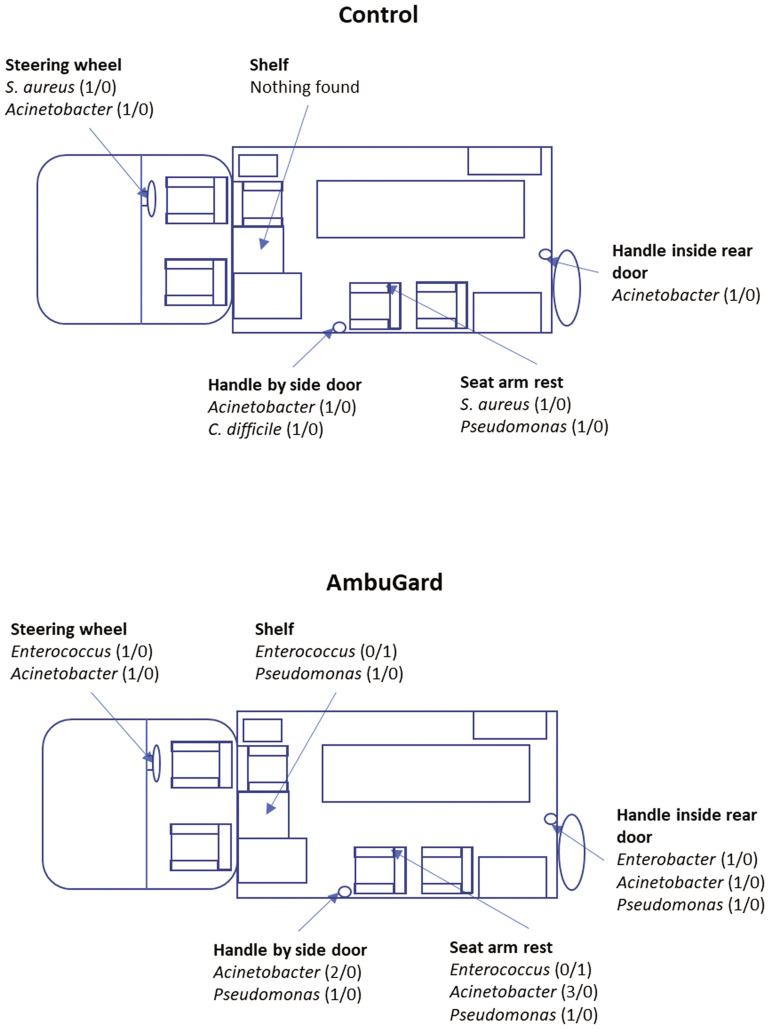
Figure 1. Pathogens shown by trial arm, location and number of vehicles where they were found pre and post cleaning.

Figure 1 displays the number and locations of vehicles in which pathogens were found on control and AmbuGard vehicles. Various CoNS and others were found on all locations pre and post cleaning.

Tables 3, 4 and 5 display the total number of CFUs of the specific pathogens (Table 3), CoNS (Table 4) and other microbes (Table 5) that were found pre and post cleaning in both study arms across all 14 vehicles. Laboratory values reported as > 100 have been included as 100 to enable the data to be summarised.

**Table 3.** Total CFUs for specified pathogens found pre and post cleaning.

	Control		AmbuGard	
	Pre clean	Post clean	Pre clean	Post clean
***Enterococcus***	0	0	1	5
***Enterobacter***	0	0	100	0
***Klebsiella***	0	0	0	0
***S. aureus***	9	0	0	0
***Acinetobacter***	9	0	191	0
***Pseudomonas***	100	0	109	0
C. difficile*	+	0	0	0

*C. difficile was reported as present or absent rather than in CFUs.

**Table 4.** Total CoNS CFUs found pre and post cleaning.

	Control		AmbuGard	
	Pre clean	Post clean	Pre clean	Post clean
**Unspecified coagulase negative staphylococci**	0	4	80	0
***Micrococcus luteus***	5	0	9	0
***S. arlettae***	0	0	1	0
***S. capitis***	102	5	16	34
***S. epidermidis***	233	228	47	26
***S. haemolyticus***	21	1	14	4
***S. hominis***	7	12	81	8
***S. pasteuri***	114	0	4	2
***S. saprophyticus***	0	1	118	2
***S. warneri***	270	1	0	3
***S. xylosus***	0	0	1	0
**Total**	749	252	364	79

**Table 5.** Total other CFUs found pre and post cleaning.

	Control		AmbuGard	
	Pre clean	Post clean	Pre clean	Post clean
***Aerococcus viridans***	22	0	57	0
***Aeromonas* sp.**	129	0	109	0
**Alpha haemolytic streptococci**	0	5	0	0
***Aspergillus fumigatus***	0	0	1	0
***Bacillus* sp.**	136	4	78	34
***Brevibacterium* sp.**	0	0	3	0
***Citrobacter gillenii***	0	0	0	0
***Clostridium perfringens***	38	0	9	1
***Curtobacterium flaccumfaciens***	0	0	3	0
***E. coli***	1	0	0	0
***Exiguobacterium* sp.**	2	0	0	0
***Kocuria palustris***	0	1	2	0
***Leclercia adecarboxylata***	0	0	0	0
***Lelliottia amnigena***	1	0	0	0
***Lichtheimia corymbifera***	1	0	0	0
***Lysinibacillus fusiformis***	1	0	0	0
***Moraxella osloensis***	0	2	0	0
**Mucoraceous mould**	0	0	1	0
***Paenibacillus amyloltyicus***	0	0	1	0
***Paenibacillus pabuli***	8	0	0	0
***Paenibacillus* sp.**	0	1	2	0
***Paenibacillus urinalis***	0	0	1	0
***Pantoea agglomerans***	19	0	149	100
***Pantoea septica***	0	0	0	0
***Pantoea* sp.**	0	0	7	0
***Rothia mucilaginosa***	0	1	0	0
***Shewanella putrefaciens***	19	0	0	0
**Total**	380	14	430	135

The cleaning staff who used AmbuGard were asked for feedback at the end of the study, using a short seven-question survey. The cleaning team returned a collective response, which is presented in Table 6.

**Table 6.** Feedback from staff who used AmbuGard.

Question	Response
How did you find using AmbuGard? (Likert scale: v. difficult, difficult, neutral, easy, v. easy)	Neutral
Did AmbuGard make any difference to the time needed for the deep-clean process? (Likert scale: much longer, longer, neutral, shorter, much shorter)	Much longer
Do you think AmbuGard improves the cleaning of the vehicle? (Yes, no, unsure)	Unsure
Do you think AmbuGard could be used by frontline crews? (Yes, no, unsure)	No
Where and how do you think would be best to use a system like AmbuGard? (Free text)	In a ventilated area
How could AmbuGard be improved? (Free text)	Without any side effects, e.g. dry mouth, headaches (mild), sore tongue
Any other comments? (Free text)	Won’t know outcome until results back

## Discussion

This exploratory study showed that 71% of included ambulances carried at least one named pathogen of interest and that all ambulances had a diverse microbial ecosystem. Normal deep cleaning and deep cleaning supplemented with AmbuGard both appear to be highly effective at removing named pathogens. Adding the AmbuGard system showed no obvious benefit over current deep-cleaning practices at this time point, and in two instances pathogens were found post cleaning on AmbuGard ambulances, which is discussed below (Table 2). Pathogens were found on all the locations swabbed, with no location standing out as overly clean or contaminated. A diverse range of other organisms, some of which are concerning and some of which may be harmless, were found on the vehicles, but deep cleaning, with or without AmbuGard, virtually eliminated these (Tables 4 and 5).

### Results in context

Ambulances are not expected to be sterile environments, but efforts must be made to reduce any potential risk to patients and staff. Pathogens have been found in ambulances in previous studies, and effectiveness of cleaning methods has been reported largely using ATP to measure effectiveness. This study adds to the limited body of literature on the type of pathogens found in ambulances and the effectiveness of cleaning, and adds an evaluation of the effectiveness of the AmbuGard dry misting system.

### Limitations and strengths

The use of laboratory analysis of swabs is a strength of this study, as other studies have used ATP swabs which do not identify which pathogens are present. Limitations include: the small number of vehicles, which was dictated by the funding available; the single time point at which swabs were taken; the non-sterile environment in which the swabbing and cleaning took place; the collective feedback from the cleaning team; and the small number of areas that were swabbed, which were all hard vehicle-mounted surfaces.

The impact of cleaning was judged by the presence and amount of CFUs of the pathogens of interest. The number of CFUs for pathogens at individual locations varied widely. Deciding how impactful a single CFU of *Enterococcus* is, or how concerned staff and patients need to be by > 100 CFU of *Enterobacter*, is challenging. In this study, a limited number of areas were swabbed and only a small amount of surface area was swabbed in each location. This limits the conclusions that can be drawn from the results. However, if these amounts of pathogens were found in high-contact and therefore presumably frequently cleaned areas, then the actual amount of these and other pathogens may be higher.

### Sources of bias

The cleaning staff involved in the study were trained on the AmbuGard then asked to apply it after their normal deep cleaning. As these staff were aware of the trial and of which locations were being swabbed, their behaviour in both arms of the trial may have changed, which could have biased the results. In addition, the cleaning staff knew when AmbuGard had been used, which may have also biased their behaviour.

The AmbuGard used unscented TriBioSan, so the researchers should not have been able to smell when it had been used. In addition, other cleaning products were used during the deep-cleaning process so any odour may have been attributed to other products, although these were not recorded. The success of blinding the researchers doing post-cleaning swabs was tested by including on the data collection form whether they thought that AmbuGard had been used. In five (36%) cases, the researcher was unsure; in the remaining nine (64%) cases, the researcher correctly identified the study arm. Although efforts to blind the researchers collecting the data were unsuccessful, the laboratory was blinded as to the intervention arm, so this was not considered a major concern.

### Generalisability

This study should be generalisable to ambulance services using regular deep cleaning, such as NEAS, but less generalisable to services using make ready crews, such as Yorkshire Ambulance Service (Mackenzie & Pilbery, 2019). Cleaning processes differ across ambulance services; however, the National Ambulance Service Infection Prevention Control (NASIPC) Group are trying to reach a consensus to standardise cleaning product.

### Controversies

Tables 2 and 3 and Figure 1 show two vehicles in which *Enterococcus* was detected post cleaning but not pre cleaning. These results could have been caused by contamination from a member of the cleaning or research team; inconsistencies in the cleaning or sampling variances, which is supported by the low colony count observed; or other reasons. This is an area that would need to be addressed in any future studies. These results go against the pattern of pathogens being eliminated by both normal deep cleaning and AmbuGard, so they may be spurious. Swabs could be taken from people and objects that had come into contact with the ambulance to identify the source of contamination if one needed to be identified.

The staff using the AmbuGard did complain of side effects that they attributed to the mist produced by the device (Table 6). This resulted in the study being suspended while a risk assessment was conducted. The study was restarted with advice to ventilate the vehicles for a period of up to 10 minutes after the AmbuGard was used. The small number of AmbuGard uses and the collective feedback make it difficult to determine how effective this measure was.

### Implications for practice and research

This study showed that pathogens associated with HAIs were found on ambulances, but it did not identify how long these had been present and cannot make any links to patient or staff impact. The presence of multiple pathogens associated with HAIs on ambulances has implications for day-to-day practice in terms of the time needed to clean an ambulance and the facilities for staff to do this. The ability of staff to conduct regular cleaning, the best methods of keeping ambulances clean and the optimal scheduling of deep cleans are all areas that could be studied further. A larger sample of vehicles would be needed to draw more robust results, and vehicles at differing points in their cleaning cycle would be needed to draw any conclusions about temporal trends in terms of pathogen load. Eco-Mist states that one potential use of AmbuGard is for sanitising ambulances in between patients, based on the short amount of time needed, which is an application that could be explored in a further study. A larger study using a system like AmbuGard in addition to daily cleaning may show more benefit than comparing against intermittent deep cleaning.

## Conclusion

Selected pathogens associated with HAIs were found on the majority of ambulances, and coagulase negative staphylococci and other microbes were found on all ambulances. Normal deep cleaning was effective, and adding AmbuGard showed no obvious improvement. This was a small study at a single point in time. Further research is needed into temporal trends, how to reduce pathogens during normal clinical duties and patient/staff impact.

## Acknowledgements

The research team would like to thank: the NE&NC AHSN for funding the study; Eco-Mist for supporting the study; the cleaning staff at Pallion without whom the study could not have happened; Michelle Jackson and Emma Burrow from NEAS R&D who supported the study; and the staff at Newcastle upon Tyne Hospital Microbiology and Virology laboratories.

The team acknowledges the support of the National Institute for Health Research Clinical Research Network (NIHR CRN).

## Author contributions

GM devised and co-led the study and drafted the manuscript. KC devised and co-led the study and reviewed the manuscript. JM contributed to the study design and analysis and reviewed the manuscript. KM supported delivery of the study and reviewed the manuscript. CC performed laboratory diagnostics and reviewed the manuscript. GM acts as the guarantor for this article.

## Conflict of interest

GM is on the editorial board of the *British Paramedic Journal*.

## Ethics

Research Ethics Committee (REC) review was not needed for this trial, as AmbuGard is a CE-marked device being used for its intended purpose. NEAS R&D and HRA approvals were secured (IRAS protocol ID 266440), and the study was adopted onto the NHS portfolio (CPMS ID 42809).

## Funding

This study was funded by a small project grant from the NE&NC Academic Health Sciences Network. Eco-Mist Biotechnics supplied the AmbuGard system and trained the cleaning staff in its use. Eco-Mist Biotechnics had no control over the trial design, data collection, data analyses, trial conclusions or dissemination.
